# Isolated Homocysteinemia Leading to Thromboembolism in Young Male with Normal Vitamin B12 and Folate Levels

**DOI:** 10.7759/cureus.1978

**Published:** 2017-12-22

**Authors:** Waleed Sadiq, Madeeha Subhan

**Affiliations:** 1 Internal Medicine, Shifa International Hospital; 2 Capital Hospital Islamabad, Ayub Teaching Hospital, Abbottabad

**Keywords:** dvt, pulmonary embolism, hyperhomocysteinemia, thrombophilia

## Abstract

Pulmonary embolism (PE) with isolated homocysteinemia is a rare disease. The diagnosis demands a proper clinical workup. Timely diagnosis can prevent complications and provide a better quality of life for the patient. We present a young man with homocysteinemia with deep vein thrombosis (DVT), pulmonary embolism, and normal vitamin B12 and folate levels despite being treated with rivaroxaban.

## Introduction

An elevated level of homocysteine in the body is known as homocysteinemia or hyperhomocysteinemia. The normal blood levels of homocysteine range from 5-15 μmol/L [1-2]. Individuals with severe hyperhomocysteinemia have homocysteine concentrations in the range of 50 to 500 ftmol/L [3]. An elevated level of homocysteine is a risk factor for arterial and venous thromboembolism [4]. There is a consistent relationship between plasma homocysteine levels and atherosclerotic vascular disease [5]. We present a nonsmoker, nonalcoholic young man diagnosed with homocysteinemia with deep vein thrombosis (DVT), pulmonary embolism (PE), and right ventricle (RV) dilation with normal vitamin B12 and folate levels.

## Case presentation

A 34-year-old, married, previously healthy man presented to our hospital outpatient department on December 7, 2017, with pain, tenderness, and swelling in his left leg for one week. The pain was sudden in onset and sharp, movement made the pain worse, and rest relieved it. The pain was localized to the lower leg and did not radiate. In addition to pain, he had a tense swelling of the lower extremity. No temperature changes were associated. This was his first episode. The patient was admitted and appropriately evaluated for DVT, including Doppler ultrasound and blood work. He was started on anticoagulation therapy with oral rivaroxaban. The patient tolerated the treatment well, and his prothrombin time and activated partial thromboplastin time were monitored. Five days after admission and receiving an anticoagulant, he developed dyspnea and hyperventilation in the morning. His arterial blood gas test showed respiratory alkalosis. Because the probability of PE was high, he was given a heparin infusion and was taken for a pulmonary angiogram, which showed a thrombus in the right pulmonary artery, involving the right upper middle and lower lobar arteries. Since sudden onset DVT and subsequent PE in a young male patient, despite being on anticoagulation, is rare, it raised a suspicion of underlying thrombophilia. A panel was ordered that came back positive for high levels of homocysteine. After the patient stabilized, he was again prescribed oral rivaroxaban. Vitamin B12, folate, and vitamin B6 levels were evaluated and found to be surprisingly normal because homocysteinemia is usually associated with vitamin B12 deficiency. He was also started on vitamin B6, vitamin B12, and folate.

Physical exam

The measurements of his legs before and after anticoagulation therapy are presented in Table 1.

**Table 1:** Physical exam of both legs before and after treatment

	Left leg (cm)	Right leg (cm)
Examination on presentation		
Knee	34	33
Midthigh	37	33
Ankle	24	24
Examination after anticoagulation		
Knee	34	33
Midthigh	34	33
Ankle	24	24

Notable results from the laboratory workup and coagulation profile are presented in Table 2 and Table 3, respectively. His homocysteine level was greater than 65 μmol/L. No other findings were remarkable.

**Table 2:** Laboratory workup including serum electrolytes and lipid profile

Serum values	
Sodium	136 mEq/L
Potassium	4.1 mEq/L
Alanine aminotransferase	83 U/L
Lipid profile	
Triglycerides	162 mg/dL
Cholesterol	108 mg/dL

**Table 3:** Coagulation profile of the patient at the time of presentation

Prothrombin time	17 sec
Activated partial thromboplastin time	34 sec

**Figure 1:**
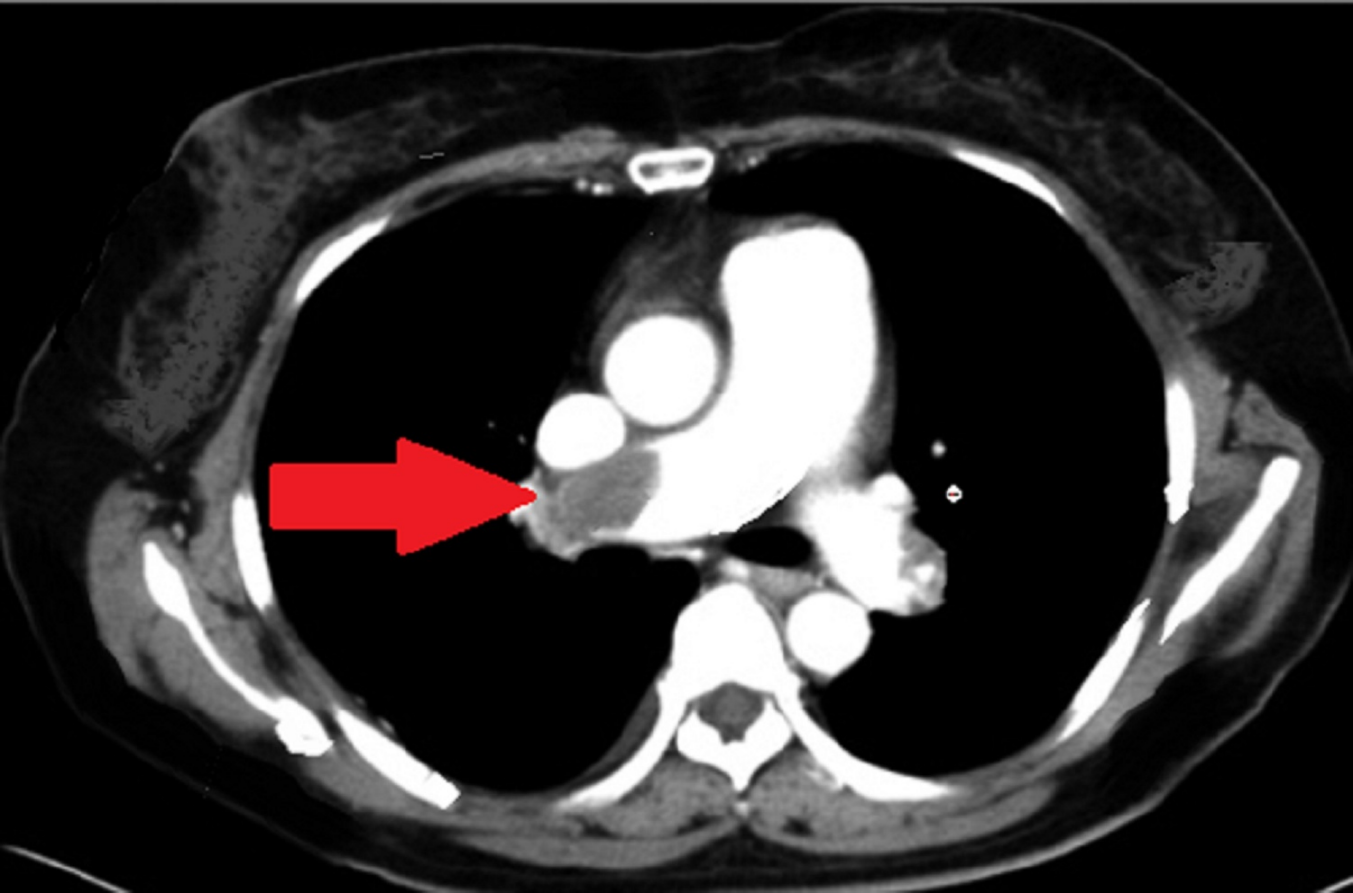
CT pulmonary angiogram showing thrombus in the right pulmonary artery CT: computed tomography

His dilated RV diameter was 27.9 mm. The Doppler ultrasound revealed thrombotic plaques in the superficial femoral vein at the midthigh level, involving the popliteal, posterior tibial, and peroneal veins. The veins were dilated and noncompressible. His perforator veins were dilated in the calf region. An electrocardiogram showed an S1Q3 pattern. The pulmonary angiogram showed thrombus in the distal right pulmonary artery, as shown in Figure 1. We also noted mild lymph node enlargement, the largest approximately 5 mm to 9 mm, in the aortopulmonary window. The anticardiolipin antibody test result was negative. D dimers were 1290 ng/mL (i.e., positive).

## Discussion

Elevated homocysteine is a strong risk factor for thrombosis and pulmonary embolism [4]. The classification of hyperhomocysteinemia is as follows: (1) moderate risk, 15 to 30 µmol/L; (2) intermediate risk, 30 to 100 µmol/L; (3) severe risk, >100 µmol/L [6]. Our patient had abnormally high homocysteine levels with normal vitamin B12 and folate levels. The patient's mean corpuscular volume was in the normal range, with no macrocytosis on peripheral film. Despite being on appropriate anticoagulation with rivaroxaban for five days, he developed PE. It is necessary to monitor a patient with homocysteinemia carefully due to the high risk of recurrence of thromboembolic events. Homocysteine is a sulfhydryl amino acid formed by the demethylation of dietary methionine [7]. Homocysteine levels in the blood are usually elevated in patients with folate deficiency because folate is required for the remethylation of homocysteine to methionine. A case-control study by Falcon et al. found that hyperhomocysteinemia was a risk factor for thrombosis in people younger than 40 years [8]. Vitamins B6 and B9 or B12 supplements, while they lower homocysteine level, do not change the risk of heart disease, stroke, or death [9].

## Conclusions

Homocysteinemia is a rare autosomal disease. Any young patient presenting with DVT should have his homocysteine level checked, and despite getting appropriate coagulation, such patient should be carefully monitored for PE, as early recognition of the problem can not only reverse but prevent PE. In some patients, vitamin B12 and folate levels can be normal, with no macrocytic anemia, but isolated homocysteinemia should be a differential diagnosis, as it can cause thromboembolism.
